# Improvement of sleep and melatonin in children with autism spectrum disorder after β‐1,3/1,6‐glucan consumption: An open‐label prospective pilot clinical study

**DOI:** 10.1002/brb3.2750

**Published:** 2022-08-22

**Authors:** Kadalraja Raghavan, Vidyasagar Devaprasad Dedeepiya, Ramesh Shankar Kandaswamy, Mangaleswaran Balamurugan, Nobunao Ikewaki, Tohru Sonoda, Gene Kurosawa, Masaru Iwasaki, Senthilkumar Preethy, Samuel JK Abraham

**Affiliations:** ^1^ Department of Paediatric Neurology Kenmax Medical Service Private Limited Madurai India; ^2^ Department of Paediatric Neurology Sarvee Integra Private Limited Chennai India; ^3^ Department of Paediatric Neurology Jesuit Antonyraj memorial Interdisciplinary Centre for Advanced Rehabilitation and Education (JAICARE) Madurai India; ^4^ Mary‐Yoshio Translational Hexagon (MYTH) Nichi‐In Centre for Regenerative Medicine (NCRM) Chennai India; ^5^ Department of Psychiatry Lincolnshire Partnership NHS Foundation Trust Lincoln United Kingdom; ^6^ Department of Neurosurgery Brain and Spine Hospital Chennai India; ^7^ Department of Medical Life Science Kyushu University of Health and Welfare Japan; ^8^ Department of Immunology Junsei Educational Institute Nobeoka Miyazaki Japan; ^9^ Department of Academic Research Support Promotion Facility, Center for Research Promotion and Support Fujita Health University Aichi Japan; ^10^ Research Wing MabGenesis KK Nagoya Japan; ^11^ Centre for Advancing Clinical Research (CACR) School of Medicine, University of Yamanashi Chuo Japan; ^12^ Fujio‐Eiji Academic Terrain (FEAT) Nichi‐In Centre for Regenerative Medicine (NCRM) Chennai India; ^13^ Antony‐ Xavier Interdisciplinary Scholastics (AXIS) GN Corporation Co. Ltd. Kofu Japan

**Keywords:** autism spectrum disorder (ASD), beta‐glucan, food supplement, melatonin, sleep

## Abstract

**Introduction:**

Poor sleep quality is a major problem in patients with autism spectrum disorder (ASD), and is attributed to low melatonin levels. Melatonin supplementation is recommended; however, its effectiveness varies. β‐Glucans have previously been shown to improve melatonin levels in animal studies. Herein, we examined the effectiveness of *Aureobasidium pullulans* (Nichi Glucan), a species of black yeast that contains beta‐1,3/1,6‐glucan, in a pilot study of children with ASD.

**Methods:**

Thirteen children (age, 2.5–13 years) with ASD were recruited for the study. The control group consisted of four patients (Gr. 1), while nine patients were classified into the treatment group (Gr. 2). Gr. 2 received 1 g of Nichi Glucan along with conventional therapy, whereas the Gr. 1 (control) patients received conventional therapy alone for 90 days. Serum melatonin levels and sleep patterns, assessed using a subjective questionnaire, were evaluated before and after treatment.

**Results:**

In Gr. 2, the average serum melatonin level increased from 238.85 ng/L preintervention to 394.72 ng/L postintervention. Eight of nine participants (88%) in Gr. 2 showed improvements in sleep pattern and quality, while no improvement was observed in the participants in Gr. 1.

**Conclusion:**

The consumption of Nichi Glucan for 90 days resulted in visible improvement in sleep quality, sleep pattern, and serum melatonin levels, which was reported for the first time by our study. A larger multicenter study is required to validate our findings.

## 1 INTRODUCTION

Sleep problems are reported in 50%−80% of children with autism spectrum disorder (ASD) (Goldman et al., 2012), and are most frequent in older children and adolescents. These problems include delayed sleep onset, shorter sleep duration, and daytime sleepiness, whereas bedtime resistance, sleep anxiety, parasomnia, and night waking are predominant in younger children (Malow et al., 2012). Sleep problems can exacerbate other features of autism, such as tantrums, aggression, self‐injury, inattention, hyperactivity, social interactions, and repetitive behaviors, adding to parental stress and negatively impacting the entire family's well‐being (Gagnon & Godbout, 2018; Goldman et al., 2012).

Melatonin, a neurohormone secreted by the pineal gland, regulates circadian rhythms, including sleep patterns, and has been shown to be released at lower levels in individuals with ASD compared to healthy individuals. Melatonin has been shown to exert a positive effect on sleep in individuals with autism by relieving anxiety, improving sensory processing, possessing anti‐nociceptive effects, and mitigating gastrointestinal (GIT) dysfunction or gut dysbiosis (Gagnon & Godbout, 2018). Indeed, a significant proportion of children with ASD suffer from chronic GIT problems such as diarrhea, constipation, and irritable bowel syndrome. These GIT symptoms are related to the cortisol response to stress and gut dysbiosis–induced chronic inflammation (Malow et al., 2012), which in turn are associated with altered melatonin levels (Malow et al., 2012). Thus, melatonin supplementation (Cortesi et al., 2012; Malow et al., 2012; Maras et al., 2018) is one of the main pharmacological approaches used to treat ASD. Clinical studies of melatonin supplementation in children with ASD have shown an improvement in sleep latency and quality (Cortesi et al., 2012; Malow et al., 2012; Maras et al., 2018), although the effectiveness varies (Gagnon & Godbout, 2018). It is also worth noting that although side effects have been reported to be minimal, melatonin has been found to be effective mostly in the short‐term treatment of sleep disorders, with clinical studies demonstrating that the positive effects decrease during follow‐up (6−12 months) (Russcher et al., 2013).

A previous study showed that when rats were fed an extract consisting of a mix of rice bran and *Sarcodon aspratus* (mushroom), which contains beta‐glucan, blood serum melatonin levels were upregulated (Costa, 2019; Dutta et al., 2021). In addition, clinical research reports have revealed the beneficial effects of Nichi Glucan, a beta‐1,3/1,6‐glucan food supplement derived from the *Aureobasidium pullulans* strain AFO‐202, on metabolic disorders (Dedeepiya et al., 2012; Ganesh et al., 2014) and cancer (Mio, 2014; Mizobuchi et al., 2008), and as a vaccine adjuvant for coronavirus disease 2019 (Ikewaki et al., 2021). In a pilot clinical study, we previously explored the effects of Nichi Glucan on sleep patterns and serum melatonin levels in children with ASD (available as preprint; Raghavan et al., 2021).

## 2 METHODS

The study was registered in India's Clinical Trial Registry CTRI (Ref no: CTRI/2020/10/028322).

URL: http://ctri.nic.in/Clinicaltrials/showallp.php?mid1 = 47623&EncHid = &userName = kenmax. URL: CTRI/2020/10/028322

The study was approved by the Institutional Ethics Committee (IEC) of Saravana Multispecialty Hospital, Madurai, India on August 24, 2019(Ref ID: GLU/2020/01).

### 2.1 Study design

The enrolled participants were clinically diagnosed with ASD by developmental pediatricians using standard assessments that involved clinical interviews incorporating the Childhood Autism Rating Scale (CARS). Overall, 18 subjects with ASD were enrolled in this prospective, open‐label pilot clinical trial, and were stratified into two groups:

Gr. 1: A control group of participants with ASD (*n* = 6) who underwent conventional treatment comprising remedial behavioral therapy and 500 mg of L‐carnosine per day.

Gr. 2: Treatment arm comprising participants (*n* = 12) who received supplementation with Nichi Glucan along with conventional treatment. Each participant consumed two sachets (0.5 g ‐ 21 mg of active ingredient) of Nichi Glucan with meals twice daily for a period of 90 days.

The participants were randomly assigned into the two groups in a 1:2 ratio.

The participants did not take any other psychiatric drugs or supplements that could affect sleep in any way.

### 2.2 Inclusion criteria

1. Age: 3–18 years
2. Sex: male or female
3. ASD criteria based on the cumulative scores obtained with the CARS scale
4. Parents willing to give consent for their children to actively participate in the study

### 2.3 Exclusion criteria

1. Age > 18 years
2. Acute general illness or any antibiotic, anti‐inflammatory, or antioxidant treatment in the 2 weeks prior to study enrolment
3. Hyperallergy to any of the investigational products
4. Presence of chronic infections

### 2.4 Outcome measures

#### 2.4.1 Primary endpoints

1. *Sleep pattern assessment by questionnaire*:

Parents and caregivers completed the survey questionnaire. Sleep problems were assessed using the children's sleep habits questionnaire (CSHQ‐A), which consisted of 22 questions (National Institute of Child Health and Development [NICHD] Study of Early Child Care and Youth Development [SECCYD]—Wisconsin), and was adapted to suit the local cultural and social conditions.

1. *Evaluation of serum melatonin*:

Melatonin levels in the serum were measured in the peripheral blood collected during the daytime, and analysis was performed using a human melatonin enzyme‐linked immunoassay Kit (BT‐LAB—Bioassay Technology Laboratory kit, China). The catalogue number was E1013Hu and the sensitivity was 2.51 ng/L.

#### 2.4.2 Secondary endpoints

Safety monitoring: The frequency and severity of adverse events and any clinically abnormal safety parameters were monitored through telephonic conversation and during the participants’ visits on days 28, 56, and 90.

#### 2.4.3 Target population for analysis

The intention‐to‐treat (ITT) analysis included all participants enrolled in the study. Per‐protocol analysis (PPS) was performed on participants who completed the entire study without dropping out.

#### 2.4.4 Method of analysis

All data were analyzed using statistics package of Excel software (Microsoft Office Excel®). Paired Student's *t*‐tests were also calculated using this package, and *p* values < .05 were considered significant.

## 3 RESULTS

### 3.1 ITT and PPS participants

Eighteen patients who met the inclusion and exclusion criteria were included in the study and the ITT analysis. One participant in the treatment group (Gr. 2) dropped out before the start of the study. Figure S1 shows a CONSORT diagram of the trial.

During the study, four subjects were lost to follow‐up: two each in Gr. 1 (one dropped out due to social problems in the family, and the other relocated to another city) and Gr. 2 (one dropped out due to social problems in the family and the other relocated to another city). After excluding these four subjects, 13 were finally included in the PPS. Their ages ranged from 2.5 to 14 years (average, 6.8 years). Though one subject was only 2.5 years, which falls out of the inclusion criteria, the investigator felt that the subject would benefit from the study, he applied for a waiver from the sponsor and notified the Independent Ethics Committee (IEC) about enrolling this subject who did not meet the inclusion criteria.

### 3.2 Primary end points

#### 3.2.1 Improvement in sleep pattern

**FIGURE 1:**
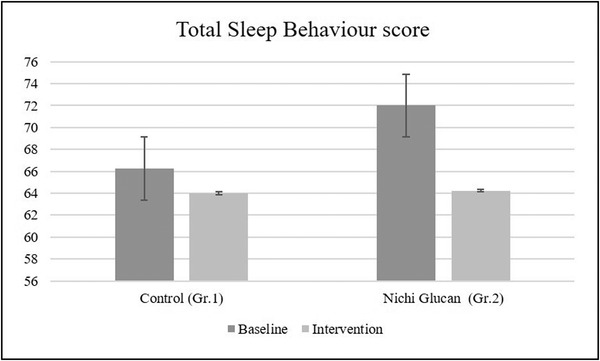
The total sleep pattern score based on the children's sleep habits questionnaire–abbreviated form (CSHQ‐A). The results suggest that the Nichi Glucan treatment resulted in a significant improvement in the sleep parameters, based on the decrease in the total score in participants in Gr. 2 (Nichi Glucan‐treated group) compared to that of those in Gr. 1 (control group). Error bars indicate standard error

In the CSHQ, a reduction in the total score was observed in Gr. 2 compared with that of Gr. 1 (Figure 1), particularly in terms of bedtime resistance and time of sleep onset (Table 1). At the baseline measurement, the mean total sleep score (range [mean ± standard deviation]) was 66.25 ± 0.5(66−67) and 72 ± 5.02(62−75) in Gr. 1 and Gr. 2, respectively. Posttreatment, the total sleep scores were 64 ± 4(58−66) and 64.22 ± 7.47(51−70) in Gr. 1 and Gr. 2, respectively. The reduction in sleep score after the intervention, which indicated an improvement in sleep behavior, was significant in Gr. 2 (*p* = .009), suggesting a significant improvement in sleep patterns with Nichi Glucan treatment. No significant differences were observed in the control group (*p* = 0.153). When the groups were compared, the total sleep score was found to be lower in Gr. 1 both pre‐ and postintervention than in Gr. 2, although the difference was not significant (*p* = .105).

**TABLE 1:** Results of the responses from the children's sleep habits questionnaire (CSHQ), which showed a reduction of the overall sleep score in Gr. 2 compared to that in Gr. 1, resulting from a decrease in the scores of bedtime resistance and time of sleep onset

Parameters	Gr.1				Gr.2			
	Baseline		End of study		Baseline		End of Study	
	Mean	SD	Mean	SD	Mean	SD	Mean	SD
Bedtime resistance	28	2	25.75	2.5	28.55	2.4	23.22	5.6
Sleep behavior	20.25	1.5	20.25	1.5	21.88	2.02	19.44	3.17
Waking during the night	6	0	6	0	6.44	1.33	5.55	1.33
Morning waking	6	0	6	0	6	1.33	6	1.33
Day‐time sleepiness	6	0	6	0	9.11	2.6	10	0

#### 3.2.2 Serum melatonin levels

**FIGURE 2:**
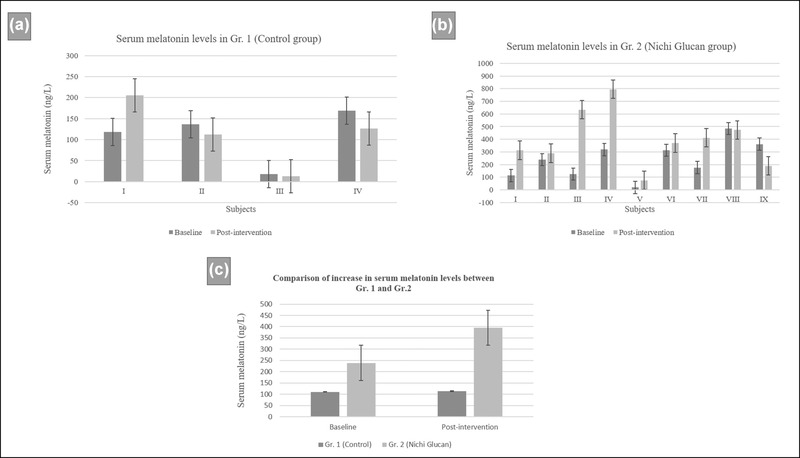
(a) Serum melatonin levels in Gr. 1 (control group) pre‐ and postintervention. (b) Gr. 2 (Nichi Glucan‐treated group). (b) The increase in serum melatonin in participants in Gr. 2 was greater than that in participants in Gr. 1. Error bars indicate standard error

Melatonin levels increased in six of eight participants in the Gr. 2 and in only one subject in the Gr. 1. On average, the serum melatonin level of the participants in Gr. 1 increased from 110.585 ng/L to 114.11 ng/L (Figure 2a), whereas those in Gr. 2 increased from 238.85 ng/L to 394.72 ng/L (Figure 2b). The increase in Gr. 2 was 2.29, compared to 1 in Gr. 1 (Figure 2c), although the difference was not significant (*p* = .06). The postintervention increase in serum melatonin level was significant in the Gr. 2 compared to that in Gr. 1 (*p* = .001).

#### 3.2.3 Secondary endpoint

##### Adverse effects

Only one patient in Gr. 2 exhibited mild adverse effects. One week after initiating Nichi Glucan supplementation, the patient showed an increase in the number of bowel movements, which resolved without treatment. No adverse effects were observed in any of the other participants.

## 4 DISCUSSION

In this open‐label clinical trial, we found that the majority of children treated with Nichi Glucan (8/9; 88%) experienced an improvement in their sleep pattern and quality of sleep, as indicated by a decrease in sleep scores. The serum melatonin levels increased to a greater extent in Gr. 2 compared to that in Gr. 1. The sleep score was also significantly decreased in Gr. 2 compared with that in Gr. 1 (Figure 1). Furthermore, only a minimal number of adverse events were observed.

This is the first study of its kind in which a nutritional supplement other than a pharmacological drug showed an ability to improve sleep patterns based on evidence from the evaluation of serum melatonin levels in children with ASD.

Sleep difficulties are a major problem in children with ASD, with individuals often reporting difficulty with sleep onset (53%), restless sleep (40%), night‐time awakening (34%), and difficulty arousing from sleep (32%) (Parvataneni et al., 2020). Poor sleep impairs emotional and daily functioning, leading to the impairment of academic/social functioning and the ability to maintain relationships. Therefore, ensuring high‐quality sleep is an essential part of therapy for children with ASD. Among the pharmacological interventions available, melatonin (Cortesi et al., 2012; Malow et al., 2012; Maras et al., 2018), antipsychotics such as olanzapine and risperidone, antidepressants such as trazodone, and alpha‐adrenergic agonists such as clonidine and guanfacine antihistamines such as trimeprazine and niaprazine, and sedatives such as clonazepam are the most commonly used medications (Ikewaki et al., 2007; Relia & Ekambaram, 2018). Melatonin supplementation remains the treatment of choice given the strong side effects associated with other interventions and considering that clinical trials have shown positive outcomes of melatonin supplementation (Ikewaki et al., 2007). Nevertheless, some reports have indicated that melatonin is more effective in the short term than in the long term, although these have mostly been conducted in cohorts of individuals without ASD (Russcher et al., 2013).

Nutritional supplements that are easy to administer and have minimal‐to‐no adverse effects are an ideal alternative to melatonin. In the current study, Nichi Glucan, which has been consumed as a food supplement for several decades (Braam et al., 2018) and has been proven to be beneficial for the treatment of diseases such as metabolic disorders and cancer (Dedeepiya et al., 2012; Ganesh et al., 2014; Mio, 2014; Mizobuchi et al., 2008), was shown to be a promising alternative to melatonin. This supplement functions to improve sleep quality and increase daytime serum melatonin levels.

It has been postulated that the etiology of low melatonin levels in children with ASD arises from melatonin deficiency in the mothers, and that melatonin exerts its effects on the embryo during neurodevelopment (Li et al., 2019). Another study reported a clear correlation between the gut microbiome profiles of children with ASD and their mothers, suggesting the importance of assessing the microbiome during the early stage of a mother's pregnancy, as well as the importance of planning personalized treatment and prevention of ASD via microbiota modulation (Li et al., 2019). Beta‐glucans have also been shown to reduce the underlying chronic inflammation associated with gut dysbiosis, as well as to help foster a healthy microbiome. This is advantageous for individuals with ASD as chronic inflammation has been shown to be associated with symptom severity (Jyonouchidoi, 2019). Thus, as the current study showed that β‐glucans can enhance melatonin and sleep quality in children with ASD, it is necessary to further investigate the ability of β‐glucans to modulate gut microbiota and reverse gut dysbiosis, which are the possible mechanisms behind altered melatonin levels (Cheng et al., 2020; Russcher et al., 2013; Shi et al., 2020), and thereby improve sleep.

This study has several limitations, which should be noted. First, the Autism Diagnostic Interview‐Revised (ADI‐R) and Autism Diagnostic Observation Schedule (ADOS) are the standard measures used to diagnose ASD. However, we used CARS for ASD diagnosis because it is the most widely used tool in low‐ and middle‐income countries (LMICs) (Samms‐Vaughan et al., 2017). The administration requirements of the ADOS and the ADI‐R, which are widely used in high‐income countries (HICs), make them less feasible for the diagnosis of ASD in LMICs. The flexible administration requirements of CARS have resulted in its wide use in both HICs and LMICs (Samms‐Vaughan et al., 2017). As India, where the study was conducted, falls under the LMIC category, we used CARS for ASD diagnosis. The other limitations of the study include the limited number, wide age range, and unequal distribution of sexes in the participants, and the differences in the number of participants between the groups. In addition, the dropout rate from this study was very high. However, this was only a pilot study, and larger randomized, multicentric clinical trials of longer duration are required to validate the results. We are planning to attain a clinically meaningful response and overcome the above‐mentioned limitations. In addition, future research should explore the possible beneficial effects of Nichi Glucan on the behavioral aspects of people with ASD, as well as other symptoms in patients with ASD.

## 5 CONCLUSIONS

In this open‐label pilot clinical study, patients with ASD showed improved sleep quality and improved levels of serum melatonin following nutritional supplementation with a beta‐1,3/1,6‐glucan (Nichi Glucan) derived from the AFO‐202 strain of black yeast *Aureobasidium pullulans*. Given that Nichi Glucan was found to be effective for improvement of sleep and other parameters in this pilot study of children with ASD, it may be worth recommending it as a supplement in such children, given that results from larger studies with longer‐term follow‐up are consistent with our findings. Further in‐depth evaluation of these mechanisms and their correlation with other neurological parameters is recommended. These findings may shed light on the development of novel solutions and drug candidates for ASD.

## FUNDING

No external funding was received for the study.

## CONFLICT OF INTEREST

Author Samuel Abraham is a shareholder in GN Corporation, Japan, which holds shares of Sophy Inc., Japan, the manufacturers of novel beta‐glucans using different strains of *Aureobasidium pullulans*; a board member in both the companies; and also an applicant to several patents of relevance to these beta‐glucans.

### PEER REVIEW

The peer review history for this article is available at https://publons.com/publon/10.1002/brb3.2750

## Supporting information

FIGURE S1 CONSORT flow diagram of the trial

## Data Availability

All data generated or analyzed during this study are included in the article (and its supplementary information files).
