# Supplementary material for: Improvement of sleep and melatonin in children with autism spectrum disorder after β‐1,3/1,6‐glucan consumption: An open‐label prospective pilot clinical study
Source: Brain Behav. 2022 Aug 22;12(9):e2750. doi: 10.1002/brb3.2750 (PMC9480952; doi:10.1002/brb3.2750)
Supplement: Supplementary file 1 — FIGURE S1 CONSORT flow diagram of the trial [file BRB3-12-e2750-s001.docx]

**Supplementary Figure 1**

**CONSORT Flow diagram of the trial**
